# Entomological Investigation Detects Dengue Virus Type 1 in *Aedes* (*Stegomyia*) *albopictus* (Skuse) during the 2015–16 Outbreak in Hawaii

**DOI:** 10.4269/ajtmh.19-0732

**Published:** 2020-02-10

**Authors:** Jeomhee M. Hasty, Gilberto E. Felix, Manuel Amador, Roberto Barrera, Gilberto S. Santiago, Lynn Nakasone, Sarah Y. Park, Steven Okoji, Eric Honda, Bernard Asuncion, Maricia Save, Jorge L. Munoz-Jordan, Stephanie Martinez-Conde, Freddy A. Medina, Stephen H. Waterman, Lyle R. Petersen, David I. Johnston, Ryan R. Hemme

**Affiliations:** 1 Hawaii State Department of Health, Honolulu, Hawaii;; 2 Division of Vector-Borne Diseases, Centers for Disease Control and Prevention, Dengue Branch, San Juan, Puerto Rico;; 3 Division of Vector-Borne Diseases, Centers for Disease Control and Prevention, Fort Collins, Colorado

## Abstract

A dengue outbreak occurred on Hawaii Island between September 2015 and March 2016. Entomological investigations were undertaken between December 2015 and February 2016 to determine which *Aedes* mosquito species were responsible for the outbreak. A total of 3,259 mosquitoes were collected using a combination of CDC autocidal gravid ovitraps, Biogents BG-Sentinel traps, and hand-nets; immature mosquitoes were collected during environmental surveys. The composition of species was *Aedes albopictus* (58%), *Aedes aegypti* (25%), *Wyeomyia mitchelli* (7%), *Aedes vexans* (5%), *Culex quinquefasciatus* (4%), and *Aedes japonicus* (1%). Adult mosquitoes were analyzed by real-time reverse transcription polymerase chain reaction (PCR) for the presence of dengue virus (DENV) RNA. Of the 185 pools of female mosquitoes tested, 15 containing *Ae. albopictus* were positive for the presence of DENV type 1 RNA. No virus was detected in pools of the remaining species. Phylogenetic analysis showed the virus strain belonged to genotype I and was closely related to strains that were circulating in the Pacific between 2008 and 2014. This is the first report of detection of DENV in *Ae. albopictus* from Hawaii.

## INTRODUCTION

Dengue is a vector-borne disease caused by four types of dengue viruses (DENV1-4, Flaviviridae, and *Flavivirus*) and is transmitted by the bite of infected *Aedes* mosquitoes, primarily *Aedes aegypti* and *Aedes albopictus*. The global burden of dengue is estimated as 390 million infections per year which is larger than previously believed.^1^ The rise in dengue incidence can be attributed to geographic range expansion of competent vectors, increased speed at which viremic humans can transport viruses across large distances, and increased urbanization.^2^

**Figure 1.**
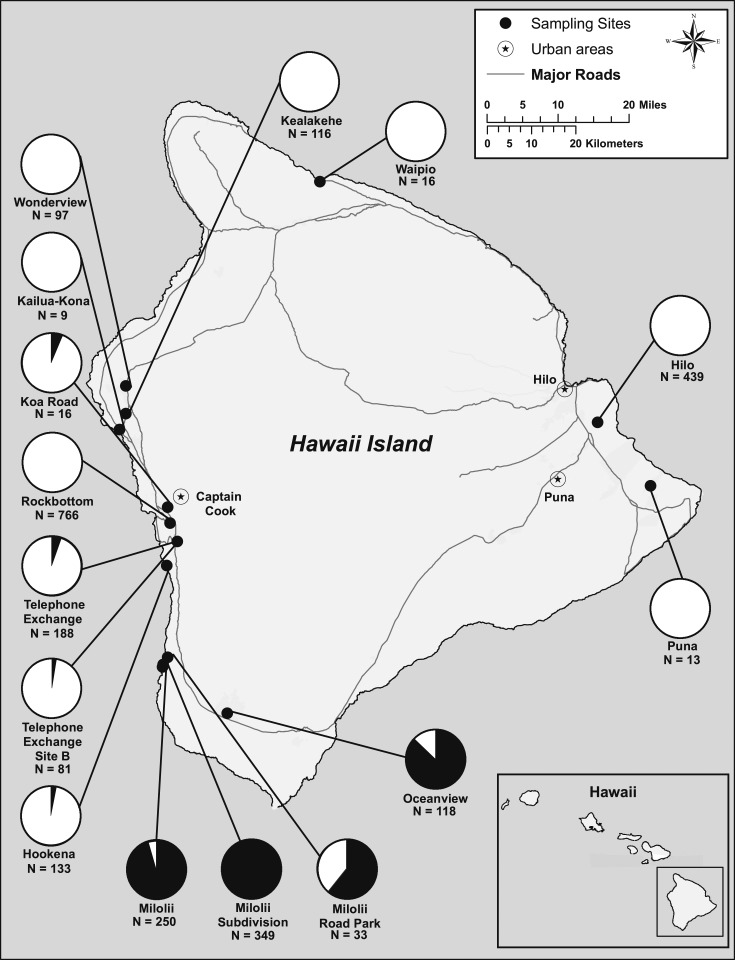
Locations and combined total *Aedes aegypti* and *Aedes albopictus* collected at each sampling site on Hawaii Island, 2015–2016. Black indicates the proportion of *Ae. aegypti* and white indicates the proportion of *Aedes albopictus*. This figure appears in color at www.ajtmh.org.

In October 2015, the Hawaii State Department of Health (HDOH) was notified of a resident who tested positive for dengue IgM and had no history of travel outside of Hawaii Island.^3^ Interviews indicated that contact with mosquitoes most likely occurred on the western side of Hawaii Island near Kona, Hawaii, and marked the first recorded case of a locally acquired infection since the 2011 outbreak on Oahu.^3,4^ The outbreak spread along populated coastal areas on the western side of the island near Kailua-Kona, Captain Cook, and Milolii, and at its peak cases were reported across the island, including Oceanview, Waipio, Hilo, and Puna (Figure 1). By the end of the outbreak in March 2016, 264 dengue cases were reported to the HDOH.

In the late 1890s, illnesses with dengue-like symptoms were reported after *Aedes* mosquitoes were introduced to Hawaii.^5,6^ Subsequent dengue outbreaks were recorded in 1903, 1912, 1943–1945, 2001, and 2011.^3,5–9^ *Aedes aegypti* was the primary vector of dengue on Hawaii during the 1943–1945 Honolulu dengue epidemic.^8^ In response, *Ae. aegypti* was eliminated from Oahu making *Ae. albopictus* the most prevalent *Aedes* species in the state.^5,8,10–13^ As part of the elimination campaign, a statewide survey conducted in the 1960s found *Ae. aegypti* had been reduced on Hawaii Island.^5,8,10,13–16^ The 2001 dengue outbreak, caused by DENV-1, was the first identified autochthonous transmission of DENV in the state in 56 years.^7^ Entomological surveys at the time found *Ae. albopictus* in 29 communities located on Oahu, Maui, Hawaii Island, Molokai, and Kauai and found *Ae. aegypti* on Hawaii Island.^7^ No DENV-positive mosquito pools were detected.^7^

Dengue epidemics vectored by *Ae. albopictus* are less common than those vectored by *Ae. aegypti* because presumably *Ae. aegypti* is better adapted to urban environments, exploits artificial containers for oviposition, and is highly anthropophagic, preferring to feed almost exclusively on humans.^17–19^ By contrast, *Ae. albopictus* will opportunistically feed on numerous mammals, including humans, dogs, cattle, and swine.^20^ *Aedes albopictus* is more common in forested or rural areas, and it will oviposit in artificial or natural (tree holes) containers when available.^21^ The objectives of this investigation were to determine the respective roles of *Ae. aegypti* and *Ae. albopictus* in the 2015–16 dengue outbreak and document the spatial distribution of these species on Hawaii Island.

## MATERIALS AND METHODS

Hawaii Island is the largest island in the State of Hawaii. Seasons are divided into summer, occurring between May and October and winter from November to April, with the winter season receiving more precipitation. The north and east side of the island is generally wetter and more humid because of orographic precipitation. This investigation took place between mid-November 2015 and mid-February 2016. Most of the mosquito trapping occurred on the western side of Hawaii Island, between Kona and Milolii, and additional collections were conducted in Waipio, Oceanview, Hilo, and Puna (Figure 1, Table 1). The locations sampled for this investigation were residential neighborhoods, including rural and sylvan areas within 800 m of clusters of laboratory-confirmed DENV cases (Tables 1 and 2). Rock Bottom was relatively rural and surrounded by macadamia nut orchards and forest compared with Wonderview and Kealakehe, which were residential neighborhoods near Kona. Hookena was a State Park on the western side of the island and Waipio was a rural community composed of single-family houses surrounded by forest. The village of Milolii, Milolii subdivision, and Oceanview were built on lava beds, did not have access to municipal water, and thus, stored water in cisterns or water storage drums/containers.

**Table 1:** Mosquito species collected using autocidal gravid ovitraps (AGOs), Biogents BG-Sentinel Traps (BGs), hand-nets (HNs), and larval collections (LCs) from sampling sites on the Big Island of Hawaii during the 2015–2016 dengue virus outbreak

Sampling site	Trap	Number of traps	Date	*Aedes aegypti*		*Ae. albopictus*		*Aedes japonicus*		*Aedes vexans*		*Culex quinquefasciatus*		*Wyeomyia mitchelli*	
				Female	Male	Female	Male	Female	Male	Female	Male	Female	Male	Female	Male
Hookena	BG^C^	2	November 17–19, 2015	3*	1	40†	15	0	0	0	0	0	0	0	0
	BG^NC^	3	December 7–12, 2015	0	0	22	9	0	0	0	0	0	1	0	0
	BG^C^	4	December 13, 2015	0	0	32†	11	0	0	0	0	0	0	0	0
Hilo	BG^C^	6	December 10–17, 2015	0	0	359*	80	0	0	150	0	11	0	0	0
Kailua-Kona	BG^C^	3	February 11–13, 2016	0	0	9*	0	0	0	0	0	0	2	0	0
Kealakehe	BG^BL^	20	January 21–February 4, 2016	0	0	4*	1	0	0	0	0	17	1	0	0
	AGO	20	January 21–February 4, 2016	0	0	78*	33	0	0	0	0	9	1	0	0
Koa Road	BG^C^	2	December 19–21, 2015	1*	0	12	3	0	0	0	0	0	0	0	0
Milolii	BG^C^	6	January 5–10, 2016	186*	53	10*	1	0	0	0	0	6	3	0	0
Milolii Road	BG^C^	3	December 22–23, 2015	47*	48	7*	3	0	0	0	0	0	0	0	0
Milolii Road Park	BG^C^	3	January 4–10, 2016	19	1	13	0	0	0	0	0	1	0	0	0
Milolii Subdivision	BG^C^	3	January 4–10, 2016	260	89	0	0	0	0	0	0	0	0	0	0
Oceanview	LC	–	January 29, 2016	51	52	5	10	0	0	0	0	0	0	0	0
Puna	BG^C^	4	December 8–9, 2015	0	0	9*	4	0	0	0	0	9	0	0	0
Rock Bottom	BG^BL^	20	December 3–17, 2015	0	0	34*	14	0	0	0	0	5	0	0	0
	AGO	20	December 3–17, 2015	0	0	473*	245	5*	0	0	0	25	6	193	0
Telephone Exchange	BG^NC^	1	November 17, 2015	1*	0	0	1	0	0	0	0	0	0	0	0
	BG^C^	1	December 3–5, 2015	1*	0	30	8	3	0	0	0	0	0	1	0
	BG^NC^	1	December 5–11, 2015	0	0	24	11	0	0	0	0	0	0	0	0
	BG^C^	1	December 13, 2015	0	0	2	3	0	0	0	0	0	0	0	0
	BG^C^	6	December 15–20, 2015	10*	0	149*	29	15*	0	0	0	2	0	40	0
Waipio	HN	–	January 26, 2016	0	0	12*	4	0	0	0	0	0	0	0	0
Wonderview	BG^BL^	20	January 21–February 4, 2016	0	0	5*	1	0	0	0	0	12	0	0	0
	AGO	20	January 21–February 4, 2016	0	0	83*	8	0	0	0	0	6	5	1	0
TOTAL	–	–	–	579	244	1,412	494	23	0	150	0	103	19	235	0

BG^C^ indicates trap was baited with carbon dioxide, BG^NC^ indicates no carbon dioxide was used, BG^BL^ indicates that a black covering was used as an visual attractant, LC indicates specimens were collected during an immature collection, and HN indicates specimens were collected with HNs for viral RNA detection.

* Indicates specimens were tested for viral RNA.

† Indicates not all of the specimens collected at this site were tested for viral RNA.

**Table 2:** Mean female captures (autocidal gravid ovitrap [AGO], trap-week; BG-Sentinel [BG] trap-day), number of pools tested, pools positive for dengue virus (DENV) RNA, and DENV infection rates from three sampling sites in Hawaii Island using AGOs and Biogents BG traps without carbon dioxide for 14 trap-nights between December 2015 and February 2016

	Mean female *Aedes albopictus*		Number of pools tested (average size)		DENV-positive pools (%)		DENV infection rate per 1,000 (95% CI)	
Sampling site	AGO (trap-week)	BG (trap-day)	AGO	BG	AGO	BG	AGO	BG
Kealakehe	0.1 (0.0–0.2)	0.3 (0.2–0.4)	2 (1)	15 (5)	0	0	–	–
Rock Bottom	0.9 (0.4–1.3)	1.6 (1.4–1.9)	7 (5)	62 (8)	2 (29)	13 (21)	62.2 (12.3, 202.4)	30.4 (17.2, 50.2)
Wonderview	0.1 (0.1–0.2)	0.3 (0.3–0.4)	3 (2)	14 (6)	0	0	–	–

At Kealakehe, Rock Bottom, and Wonderview, 20 CDC autocidal gravid ovitraps (AGO) and 20 Biogents BG-Sentinel (BG) traps with black covers and without carbon dioxide (CO_2_) were paired and placed on opposite sides of houses within a 200-m radius of the case-patient (Table 1).^22^ Trapping at the three locations was conducted for 14 days. At Hilo, Telephone Exchange, and Milolii, BG traps using CO_2_ lures without black covers were deployed for 7 days (Table 1). BG-Sentinel traps were monitored daily and AGO traps were checked every 3–4 days. Additional BG traps without black covers that varied in use of CO_2_ were placed in areas where DENV or the presence of *Ae. aegypti* was suspected, and informal surveys of aquatic habitats were performed at Waipio and Oceanview (Table 1).

Mosquito collections were transported on ice to the laboratory and were killed by freezing. All mosquitoes were sexed and identified to species. Female *Ae. aegypti*, *Ae. albopictus*, and *Aedes japonicus* were placed in 2-mL vials containing not more than 10 female mosquitoes and stored at −20°C until they could be shipped to the CDC Dengue Branch where they were stored at −80°C until tested for viral RNA.

Mosquito pools were suspended in tissue culture media and macerated using the Qiagen TissueLyser II instrument. Viral RNA was extracted and detected by dengue type-specific real-time reverse transcription PCR as described by Santiago et al.^23^ PCR reactions with cycle threshold values below 37 were considered positive. Maximum likelihood estimates of the minimum infection rate for *Ae. albopictus* were calculated using PooledInfRate version 4.0 (Fort Collins, CO).^24^

The envelope glycoprotein coding region (E gene) of DENV-1 was sequenced directly from the mosquito pool macerates and nine human clinical serum specimens that tested positive by PCR. The target region was amplified using serotype-specific primers and the resulting amplicon (1,743 bp) was sequenced using the Sanger bidirectional method from eight bidirectional and overlapping sequencing reactions using Applied BioSystems (Foster City, CA) BigDye Terminator v. 3.1 sequencing kits. Sequences were obtained directly from the original samples; virus isolation was not attempted. A total of 29 DENV-1 E gene sequences were obtained, including from 12 *Ae. albopictus* pools collected during the 2015 outbreak, nine clinical serum specimens from symptomatic humans collected in the same region during the same outbreak by the HDOH, and eight clinical serum specimens from symptomatic humans collected during the 2001 DENV-1 outbreak.^7^ To understand the genetic relatedness of these sequences in context with the South Pacific, we reconstructed a Bayesian maximum clade credibility phylogenetic tree with the 29 sequences obtained from this study and 42 sequences obtained from GenBank using BEAST v. 1.8.4. Parameters for BEAST (Edinburgh, UK) include 30 million MCMC, time of the most recent common ancestor for the 2015 monophyletic lineage, and a strict molecular clock to achieve acceptable statistical values (effective sample size > 200).

## RESULTS

We collected six species of mosquito from three genera; the most common mosquito species collected was *Ae. albopictus*, followed by *Ae. aegypti*, *Wyeomyia mitchelli*, *Aedes vexans*, *Culex quinquefasciatus*, and *Ae. japonicus* (Table 1). *Aedes albopictus* was collected at all 15 sampled sites and was the only or most abundant species at 12 of the sites, whereas *Ae. aegypti* was found at five sites and was most abundant on the southwest side of Hawaii Island near the Milolii and Oceanview sites (Figure 1).

In total, we screened 185 pools of female mosquitoes for the presence of DENV RNA of which 135 were *Ae. albopictus*, 38 were *Ae. aegypti*, and 12 were *Ae. japonicus*. We detected viral RNA for DENV-1 in 15 pools of *Ae. albopictus* from Rock Bottom, two of which were from pools using AGOs and 13 were pools from BG traps. Infection rates at Rock Bottom were 62.2 per 1,000 and 30.4 per 1,000 for AGO and BG traps, respectively (Table 2). No virus-positive pools were detected in pools of *Ae. aegypti* or *Ae. albopictus* collected in Telephone Exchange, Hilo, or Milolii (Table 3).

**Table 3:** Mean female captures per trap-day, number of pools tested, and pools positive for dengue virus (DENV) RNA at sampling sites in Hawaii Island using Biogents BG-Sentinel traps baited carbon dioxide for 7 trap-nights between December 2015 and January 2016

	Mean female per trap-day		Number of pools tested (avg. size)		DENV-positive pools (%)	
Sampling site	*Ae. aegypti*	*Ae. albopictus*	*Ae. aegypti*	*Ae. albopictus*	*Ae. aegypti*	*Ae. albopictus*
Telephone Exchange	0.2 (0.1–0.1)	3.4 (2.5–4.6)	6 (2)	16 (10)	0	0
Hilo	0	8.6 (4.6–12.6)	0	38 (9)	0	0
Milolii	4.4 (3.0–5.8)	0.2 (0.1–0.5)	22 (8)	5 (2)	0	0

*Ae. aegypti* = *Aedes aegypti*; *Ae. albopictus* = *Aedes albopictus*.

**Figure 2.**
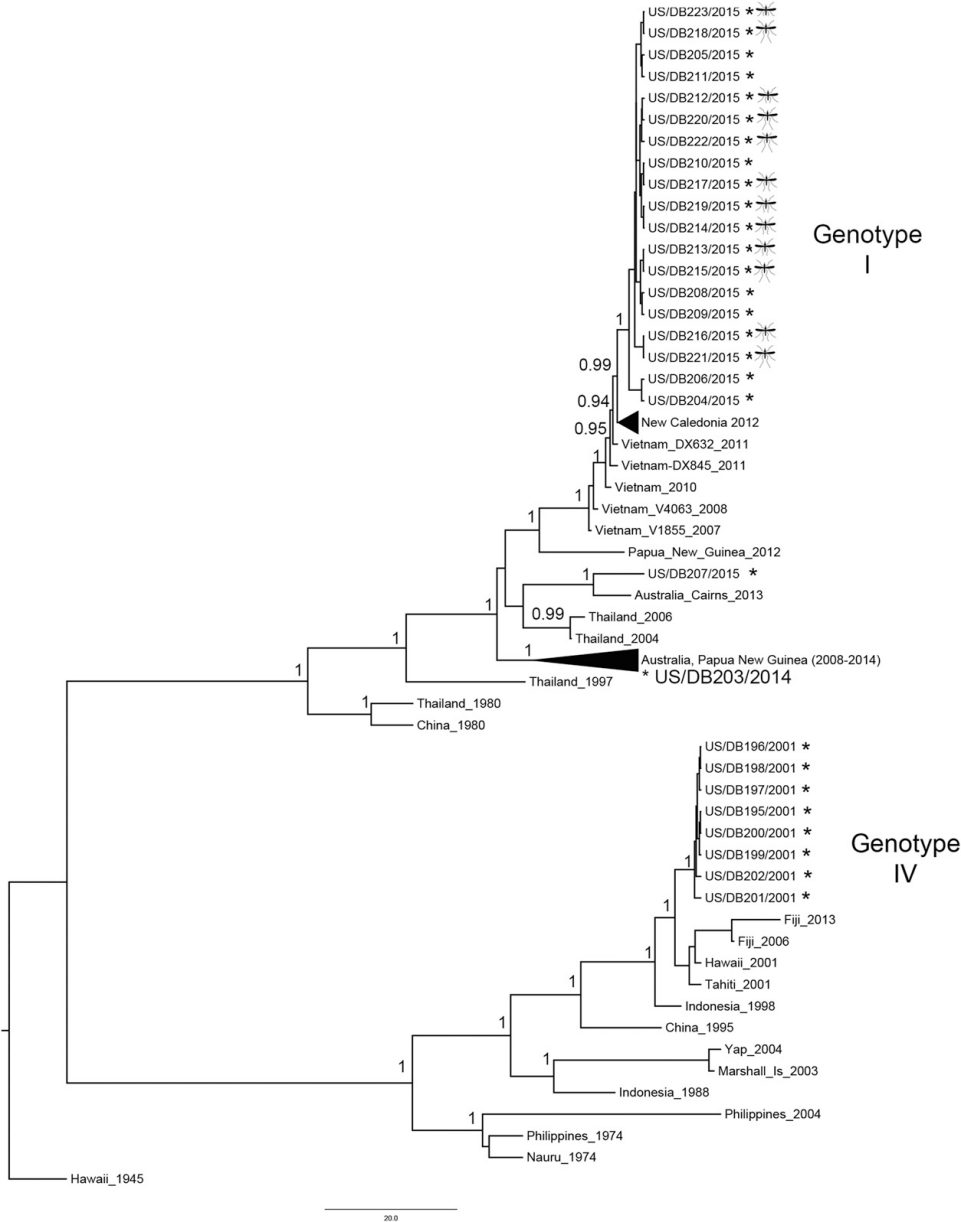
Genetic relatedness of Hawaii dengue virus (DENV-1) strains. Bayesian phylogenetic reconstruction using a maximum clade credibility tree that includes 71 DENV-1 E gene (1,485 bp) sequences and corresponds to the complete gene sequence. Posterior probabilities of major tree nodes are shown. (*) next to taxon label indicates the sequence obtained during the 2015–2016 dengue outbreak on Hawaii Island. Sequences without (*) were obtained from GenBank. Mosquito icon next to taxa label indicates sequences derived from *Aedes albopictus* hosts. Sequences without the mosquito icon were derived from human hosts.

In general, all the 21 sequences obtained from the 2015 outbreak grouped together as a monophyletic lineage and belonged to genotype I, including those derived from mosquito macerates and human specimens (Figure 2, Supplemental Table 1). No phylogenetic difference was detected between mosquito and human host sequences. Two 2015 sequences grouped separately: sequence US/DB207/2015 grouped together with a 2013 Australian (Cairns) sequence and sequence US/DB203/2014 grouped with a cluster of sequences that circulated in Australia and Papua New Guinea between 2008 and 2014. Time of the most recent common ancestor suggests that the Hawaiian 2015 lineage emerged in the region by 2013 (1.4–3.36 years 95% highest posterior density) and is closely related to sequences from New Caledonia with contemporary circulation. However, all eight sequences from 2001 grouped separately and belonged to genotype IV.

## DISCUSSION

During the 2015–16 DENV outbreak on Hawaii Island, *Ae. albopictus* was found at all sampled sites where laboratory-confirmed case-patients resided, and RNA of DENV-1 was detected by RT-PCR from pools of *Ae. albopictus* collected at Rock Bottom. Phylogenetic analysis showed the virus identified from *Ae. albopictus* and human specimens belonged to genotype I and were monophyletic. In addition, extensive mosquito trapping near clusters of DENV-confirmed case-patients in Wonderview, Kealakehe, and Hilo detected *Ae. albopictus*, but no *Ae. aegypti*. These results indicated that *Ae. albopictus* was the primary vector responsible for transmitting DENV on Hawaii Island during the 2015–16 outbreak. This is the first entomological investigation to detect DENV in *Ae. albopictus* in the state of Hawaii, confirming its importance in DENV transmission on the islands of Hawaii.

We are unable to discount the possibility that *Ae. aegypti* was involved at some foci. *Aedes aegypti* was found at six sites where dengue case-patients resided, three of which were clustered near the town of Milolii. Most mosquitoes collected at those locations (94%) were *Ae. aegypti* (*N* = 747; Figure 1). It is significant that *Ae. aegypti* was dominant in communities that relied on water storage for domestic use and for drinking. Furthermore, entomological investigations collected adult *Ae. aegypti* and *Ae. albopictus* in Hookena Beach Park, which was identified as a possible site of exposure during the early phases of the outbreak.^3^ Not all mosquito specimens were tested for viral RNA, as those collected in neighborhoods where no recent DENV cases were reported would have been less likely to be infected with DENV. Consequently, we may have not detected some virus-positive specimens because of timing of the investigation. Furthermore, sampling at Rock Bottom was conducted in December, concurrent with active transmission in humans, whereas in Kealakehe and Wonderview, specimens were collected in January, 1–2 weeks after the case-patients were reported to the HDOH. Finally, not all our investigations could be completed before vector control activities were conducted in Milolii, Telephone Exchange, and Hookena, which might have affected our findings.

*Aedes albopictus* was the vector responsible for transmitting virus in the 2001 and 2015 dengue outbreaks in Hawaii, and the outbreaks were caused by separate virus introductions (Figure 2). It has been suggested that DENV outbreaks transmitted by *Ae. albopictus* are less explosive and tend to smolder as was observed during the 2015 outbreak.^17,25,26^ However, reports of dengue outbreaks in China show that *Ae. albopictus* can drive large epidemics, suggesting that outcomes of outbreaks are in part a result of complex interactions between mosquito and human behaviors, and demographics.^27–31^ For example, during this outbreak on Hawaii Island, dengue transmission might have been slowed because of host feeding behaviors of *Ae. albopictus* (wide range of hosts, exophagy) and low population density that limited human–mosquito interactions compared with areas with *Ae. aegypti* or higher densities of humans.^31^ Recent population growth, demographic shifts to urban areas, and its prominence as an important destination and hub in the Pacific for civilians and the U.S. military may alter the epidemiology of future outbreaks.^27,32–34^ The presence of two competent vectors and imported cases of arbovirus infection pose continued risk to the residents of Hawaii, highlighting the need for rigorous monitoring and surveillance of virus and vectors.

In preparation for future arbovirus introductions, we recommend that Hawaii implement standardized surveillance programs and continue to build vector control infrastructure targeting *Ae. aegypti* and *Ae. albopictus*.^35^ Special attention should be given to targeting aquatic habitats of immature *Ae. albopictus*, which are often cryptic, and the fine-scale mapping of the occurrence and abundances of *Ae. aegypti* and *Ae. albopictus*, including their relative abundance. Local vector populations should be screened for resistance to first line of defense insecticides, and we suggest that the suitability and acceptability of novel vector control interventions in Hawaii be assessed.

## Supplemental table

Supplemental materials

## References

[1] Bhatt S 2013 The global distribution and burden of dengue. Nature 496: 504–507. 23563266 10.1038/nature12060 PMC3651993

[2] Gubler DJ, 2011 Dengue, urbanization and globalization: the unholy trinity of the 21(st) century. Trop Med Health 39: 3–11. 10.2149/tmh.2011-S05 PMC3317603 22500131

[3] Johnston D Viray M Ushiroda J Whelen AC Sciulli R Gose R Lee R Honda E Park SY; Hawaii Dengue Response Team, 2016 Notes from the field: outbreak of locally acquired cases of dengue fever–Hawaii, 2015. MMWR Morb Mortal Wkly Rep 65: 34–35. 26796994 10.15585/mmwr.mm6502a4

[4] Hawaii Department of Health, 2016 Dengue Outbreak 2015–16. Available at: http://health.hawaii.gov/docd/dengue-outbreak-2015/. Accessed January 23, 2018.

[5] Gilbertson WE, 1945 Sanitary aspects of the control of the 1943–1944 epidemic of dengue fever in Honolulu. Am J Public Health Nations Health 35: 261–270. 18016137 10.2105/ajph.35.3.261 PMC1625316

[6] Gubler DJ, 1998 Dengue and dengue hemorrhagic fever: its history and resurgence as a global health problem. Gubler DJ Kuni G, eds. Dengue and Dengue Hemorrhagic fever. London, United Kingdom: CAB International, 1–22.

[7] Effler PV 2005 Dengue fever, Hawaii, 2001–2002. Emerg Infect Dis 11: 742–749. 15890132 10.3201/eid1105.041063 PMC3320380

[8] Usinger RL, 1944 Entomological phases of the recent dengue epidemic in Honolulu. Public Health Rep 59: 423–430.

[9] Wilson GW, 1904 Epidemic of dengue in the territory of Hawaii during 1903. Public Health Rep 19: 67–70.

[10] Winchester JC Kapan DD, 2013 History of *Aedes* mosquitoes in Hawaii. J Am Mosq Control Assoc 29: 154–163. 23923330 10.2987/12-6292R.1

[11] Van Dine DL, 1904 Mosquitoes in Hawaii. Honolulu, HI: United States Department of Agriculture, 1–30.

[12] Wilbar CL Jr., 1947 Control of dengue in Hawaii. Am J Public Health Nations Health 37: 663–674. 20242031

[13] Wells CJ (US Department of Health, Education, and Welfare, National Communicable Disease Center), 1968. *Analysis of* Aedes aegypti *Eradication Program: State of Hawaii*. Atlanta, GA: CDC.

[14] Hayes JM 2006 Risk factors for infection during a dengue-1 outbreak in Maui, Hawaii, 2001. Trans R Soc Trop Med Hyg 100: 559–566. 16356519 10.1016/j.trstmh.2005.08.013

[15] Hess AD (US Department of Health, Education, and Welfare, Communicable Disease Center), 1957. *A Preliminary Appraisal of the Mosquito Control Program in the Territory of Hawaii*. Atlanta, GA: CDC, 1–70.

[16] Schliessmann DJ, 1967 Initiation of the *Aedes aegypti* eradication programme of the USA. Bull World Health Organ 36: 604–609. 5299461 PMC2476437

[17] Lambrechts L Scott TW Gubler DJ, 2010 Consequences of the expanding global distribution of *Aedes albopictus* for dengue virus transmission. PLoS Negl Trop Dis 4: e646. 20520794 10.1371/journal.pntd.0000646 PMC2876112

[18] Carvalho FD Moreira LA, 2017 Why is *Aedes aegypti* Linnaeus so successful as a species? Neotrop Entomol 46: 243–255. 28401481 10.1007/s13744-017-0520-4

[19] Harrington LC Edman JD Scott TW, 2001 Why do female *Aedes aegypti* (Diptera: Culicidae) feed preferentially and frequently on human blood? J Med Entomol 38: 411–422. 11372967 10.1603/0022-2585-38.3.411

[20] Tempelis CH Hayes RO Hess AD Reeves WC, 1970 Blood-feeding habits of four species of mosquito found in Hawaii. Am J Trop Med Hyg 19: 335–341. 5443081 10.4269/ajtmh.1970.19.335

[21] Hawley WA, 1988 The biology of *Aedes albopictus*. J Am Mosq Control Assoc 4: 1–39. 3068349

[22] Barrera R Mackay AJ Amador M, 2013 An improved trap to capture adult container-inhabiting mosquitoes. J Am Mosq Control Assoc 29: 358–368. 24551969 10.2987/13-6343.1 PMC4631063

[23] Santiago GA Vergne E Quiles Y Cosme J Vazquez J Medina JF Medina F Colon C Margolis H Munoz-Jordan JL, 2013 Analytical and clinical performance of the CDC real time RT-PCR assay for detection and typing of dengue virus. PLoS Negl Trop Dis 7: e2311. 23875046 10.1371/journal.pntd.0002311 PMC3708876

[24] Biggerstaff BJ, 2009 PooledInRate, Version 4.0: An Excel® Add-In to Compute Infection Rates from Pooled Data. Fort Collins, Colorado: Centers for Disease Control and Prevention.

[25] Issack MI Pursem VN Barkham TMS Ng LC Inoue M Manraj SS, 2010 Reemergence of dengue in Mauritius. Emerg Infect Dis 16: 716–718. 20350397 10.3201/eid1604.091582 PMC3321960

[26] Zhang FC 2014 Severe dengue outbreak in Yunnan, China, 2013. Int J Infect Dis 27: 4–6. 25107464 10.1016/j.ijid.2014.03.1392

[27] Feldstein LR Brownstein JS Brady OJ Hay SI Johansson MA, 2015 Dengue on islands: a Bayesian approach to understanding the global ecology of dengue viruses. Trans R Soc Trop Med Hyg 109: 303–312. 25771261 10.1093/trstmh/trv012 PMC4401210

[28] Kobayashi D, 2018 Dengue virus infection in *Aedes albopictus* during the 2014 autochthonous dengue outbreak in Tokyo metropolis, Japan. Am J Trop Med Hyg 98: 1460–1468. 29557338 10.4269/ajtmh.17-0954 PMC5953391

[29] Kutsuna S 2015 Autochthonous dengue fever, Tokyo, Japan, 2014. Emerg Infect Dis 21: 517–520. 25695200 10.3201/eid2103.141662 PMC4344289

[30] Luo L Jiang LY Xiao XC Di B Jing QL Wang SY Tang JL Wang M Tang XP Yang ZC, 2017 The dengue preface to endemic in mainland China: the historical largest outbreak by *Aedes albopictus* in Guangzhou, 2014. Infect Dis Poverty 6: 148. 28934991 10.1186/s40249-017-0352-9 PMC5609019

[31] Reiter P 2003 Texas lifestyle limits transmission of dengue virus. Emerg Infect Dis 9: 86–89. 12533286 10.3201/eid0901.020220 PMC2873752

[32] United States Census Bureau, 2012 Population and housing unit counts CPH-2-13, Hawaii. Bureau USC, ed. 2010 Census of Population and Housing. Washington, DC: U.S. Government Priniting Office.

[33] Hasty JM Yang P Oshiro P Nakasone L Whelen C, 2015 Mosquito surveillance program using ovitraps detected *Aedes aegypti* at the Honolulu International Airport in 2012. Proc Hawaii Entomol Soc 47: 1–11.

[34] Hawaii Department of Transportation, 2019 The State of Hawaii Airport Activity Statistics by Calendar Year. Honolulu, HI: Hawaii Department of Transportation, Airports Planning Office, Honolulu International Airport.

[35] Centers for Disease Control and Prevention, 2017 Surveillance and Control of *Aedes aegypti* and *Aedes albopictus* in the United States. Available at: https://www.cdc.gov/chikungunya/pdfs/surveillance-and-control-of-aedes-aegypti-and-aedes-albopictus-us.pdf. Accessed April 23, 2019.

