# Supplementary material for: Entomological Investigation Detects Dengue Virus Type 1 in Aedes (Stegomyia) albopictus (Skuse) during the 2015–16 Outbreak in Hawaii
Source: Am J Trop Med Hyg. 2020 Feb 10;102(4):869–75. doi: 10.4269/ajtmh.19-0732 (PMC7124917; doi:10.4269/ajtmh.19-0732)
Supplement: Supplementary file 1 [file tpmd190732.SD1.pdf]

The following are supplemental materials and will be published online only

Supplementary Table 1. GenBank accession numbers

Taxa label	Serotype	Country	Collection Date	Host	Genotype	Accession #
US/ DB195/2001	1	Hawaii	2001	human	IV	MH634394
US/ DB196/2001	1	Hawaii	2001	human	IV	MH634395
US/ DB197/2001	1	Hawaii	2001	human	IV	MH634396
US/ DB198/2001	1	Hawaii	2001	human	IV	MH634397
US/ DB199/2001	1	Hawaii	2001	human	IV	MH634398
US/ DB200/2001	1	Hawaii	2001	human	IV	MH634399
US/ DB201/2001	1	Hawaii	2001	human	IV	MH634400
US/ DB202/2001	1	Hawaii	2001	human	IV	MH634401
US/ DB203/2014	1	Hawaii	2014	human	I	MH634402
US/ DB204/2015	1	Hawaii	2015	human	I	MH634403
US/ DB205/2015	1	Hawaii	2015	human	I	MH634404
US/ DB206/2015	1	Hawaii	2015	human	I	MH634405
US/ DB207/2015	1	Hawaii	2015	human	I	MH634406
US/ DB208/2015	1	Hawaii	2015	human	I	MH634407
US/ DB209/2015	1	Hawaii	2015	human	I	MH634408
US/ DB210/2015	1	Hawaii	2015	human	I	MH634409
US/ DB211/2015	1	Hawaii	2015	human	I	MH634410
US/ DB212/2015	1	Hawaii	2015	mosquito	I	MH634411
US/ DB213/2015	1	Hawaii	2015	mosquito	I	MH634412
US/ DB214/2015	1	Hawaii	2015	mosquito	I	MH634413
US/ DB215/2015	1	Hawaii	2015	mosquito	I	MH634414
US/ DB216/2015	1	Hawaii	2015	mosquito	I	MH634415
US/ DB217/2015	1	Hawaii	2015	mosquito	I	MH634416

US/ DB218/2015	1	Hawaii	2015	mosquito	I	MH63441 7
US/ DB219/2015	1	Hawaii	2015	mosquito	I	MH63441 8
US/ DB220/2015	1	Hawaii	2015	mosquito	I	MH63441 9
US/ DB221/2015	1	Hawaii	2015	mosquito	I	MH63442 0
US/ DB222/2015	1	Hawaii	2015	mosquito	I	MH63442 1
US/ DB223/2015	1	Hawaii	2015	mosquito	I	MH63442 2
